# Land Use Regression Modelling of Outdoor NO_2_ and PM_2.5_ Concentrations in Three Low Income Areas in the Western Cape Province, South Africa

**DOI:** 10.3390/ijerph15071452

**Published:** 2018-07-10

**Authors:** Apolline Saucy, Martin Röösli, Nino Künzli, Ming-Yi Tsai, Chloé Sieber, Toyib Olaniyan, Roslynn Baatjies, Mohamed Jeebhay, Mark Davey, Benjamin Flückiger, Rajen N. Naidoo, Mohammed Aqiel Dalvie, Mahnaz Badpa, Kees de Hoogh

**Affiliations:** 1 Department of Epidemiology and Public Health, Swiss Tropical and Public Health Institute (Swiss TPH), Socinstrasse 57, CH-4002 Basel, Switzerland; apolline.saucy@swisstph.ch (A.S.); nino.kuenzli@swisstph.ch (N.K.); chloe.sieber@gmail.com (C.S.); medavey@gmail.com (M.D.); benjamin.flueckiger@swisstph.ch (B.F.); mahnaz.badpa@stud.unibas.ch (M.B.); c.dehoogh@unibas.ch (K.d.H.); 2 Faculty of Science, University of Basel, CH-4003 Basel, Switzerland; 3 Environmental and Occupational Health Sciences, University of Washington, Seattle, WA 98195 USA; mytsai@u.washington.edu; 4 Centre for Environmental and Occupational Health Research, School of Public Health and Family Medicine, University of Cape Town, Rondebosch, 7700 Cape Town, South Africa; olaniyanolan@gmail.com (T.O.); mohamed.jeebhay@uct.ac.za (M.J.); aqiel.dalvie@uct.ac.za (M.A.D.); 5 Department of Environmental and Occupational Studies, Cape Peninsula University of Technology (CPUT), 8001 Cape Town, South Africa; baatjiesr@cput.ac.za; 6 Discipline of Occupational and Environmental Health, School of Nursing and Public Health, University of KwaZulu-Natal, 4041 Durban, South Africa; naidoon@ukzn.ac.za

**Keywords:** air pollution, informal settlements, modelling, environmental exposure, exposure assessment, land use regression, nitrogen dioxide, particulate matter, South Africa, Western Cape

## Abstract

Air pollution can cause many adverse health outcomes, including cardiovascular and respiratory disorders. Land use regression (LUR) models are frequently used to describe small-scale spatial variation in air pollution levels based on measurements and geographical predictors. They are particularly suitable in resource limited settings and can help to inform communities, industries, and policy makers. Weekly measurements of NO_2_ and PM_2.5_ were performed in three informal areas of the Western Cape in the warm and cold seasons 2015–2016. Seasonal means were calculated using routinely monitored pollution data. Six LUR models were developed (four seasonal and two annual) using a supervised stepwise land-use-regression method. The models were validated using leave-one-out-cross-validation and tested for spatial autocorrelation. Annual measured mean NO_2_ and PM_2.5_ were 22.1 μg/m^3^ and 10.2 μg/m^3^, respectively. The NO_2_ models for the warm season, cold season, and overall year explained 62%, 77%, and 76% of the variance (R^2^). The PM_2.5_ annual models had lower explanatory power (R^2^ = 0.36, 0.29, and 0.29). The best predictors for NO_2_ were traffic related variables (major roads, bus routes). Local sources such as grills and waste burning sites appeared to be good predictors for PM_2.5_, together with population density. This study demonstrates that land-use-regression modelling for NO_2_ can be successfully applied to informal peri-urban settlements in South Africa using similar predictor variables to those performed in Europe and North America. Explanatory power for PM_2.5_ models is lower due to lower spatial variability and the possible impact of local transient sources. The study was able to provide NO_2_ and PM_2.5_ seasonal exposure estimates and maps for further health studies.

## 1. Introduction

Intra-urban air pollution, particularly traffic-related air pollution, has been associated with adverse health effects in children and adults, such as cardiovascular and respiratory disorders as well as overall mortality [1]. The World Health Organization (WHO) estimates that air pollution is responsible for approximately 7 million deaths worldwide every year [2,3]. In 2012, ambient air pollution from particulate matter contributed to about 3 million deaths and 85 million disability adjusted life years [4] globally, of which 600,000 deaths occurred yearly on the African continent [5]. Accurate and regular air quality monitoring is necessary to evaluate air quality to determine exceedances, identify potential sources, improve control, and advise policy makers [5]. In South Africa, air quality is monitored on a regular basis in several cities that conform to the Air Quality Management (AQM) and introduced in the Western Cape by the Department of Environmental Affairs as a measure for air quality control and planning [6]. The first phase of this plan reported generally good air quality. However, high spatial heterogeneity was reported with poor air quality at times, especially in relation to industrial areas, high traffic conditions, and low income residential areas [6]. A later report highlighted similar findings with generally limited nitrogen dioxide (NO_2_) and particulate matter (PM_10_) (PM_10_ refers to all particles smaller than 10 μm diameter. PM_2.5_ refers to particles smaller than 2.5 μm diameter.) levels in different areas between 2011 and 2015 (daily values below 200 μg/m^3^ for NO_2_ and 75 μg/m^3^ for PM_10_) and some daily excesses for small periods of time observed in Khayelitsha, up to 400 μg/m^3^ for NO_2_, mainly due to transient sources located close to the measurement station [7].

Both short-and long-term health effects of ambient air pollution are well known [8,9] and recent studies confirm these associations also at levels of air pollution below those recommended by WHO [2]. A European study demonstrated a significant increase of natural death associated with each increase of 5 μg/m^3^ in PM_2.5_ [10]. A recent review from the WHO highlighted the association between low NO_2_ exposure and respiratory and cardiovascular mortality. Although the effect of NO_2_ exposure alone is difficult to assess as it often appears together with high concentrations of other traffic-related pollutants, the WHO considers NO_2_—like PM—as an appropriate marker of air pollution as a basis for assessing health impacts [9,10]. However, few air pollution health studies have been performed in Africa, where air pollutant mixtures and susceptibility of the population may differ from other continents. For the conduct of epidemiological studies, high resolution air pollution models are required to characterize spatiotemporal differences in air pollution exposure and to accurately assess long-term air pollution exposure over large populations [11].

The land use regression (LUR) method, which is frequently used to model air pollution exposures, is able to describe small-scale spatial variation in air pollution levels based on meteorological and geographical predictor variables. The method has been widely used in Europe and North America [12,13,14], but less so in African countries, even though it offers an affordable way to model the spatial distribution of urban air pollution since these methods do not need extensive emission inventories like dispersion models. Furthermore, contributions from informal emission sources such as open waste burning are implicitly considered in LUR models. A study from 2015 applied LUR modelling in Africa to investigate the spatial variation of NO_2_ in Mauritania [15]. Recently, Muttoo et al. used LUR to predict NOX levels in Durban, South Africa [16]. The studies demonstrated that the same method as used in Western countries settings can be applied in African towns and provide consistent models and predictions.

This study is part of an epidemiological study investigating the effect of different ambient air pollutants on asthma among pupils enrolled in primary schools in or close to informal settlements in the Western Cape, South Africa [17]. The aim of this study was to characterize and model the spatial distribution of NO_2_ and PM_2.5_ concentrations in three informal settlement areas in the urban Western Cape, South Africa. The models were used to predict annual and seasonal PM_2.5_ and NO_2_ exposures at the home address of the study participants. Additionally, the study will contribute to a better understanding of the spatial distribution of air pollution in similar urban settings of the Western Cape and provide information on air pollution exposure levels for further research as well as for public health policies in the Western Cape Province.

## 2. Materials and Methods

### 2.1. Study Area

**Figure 1:**
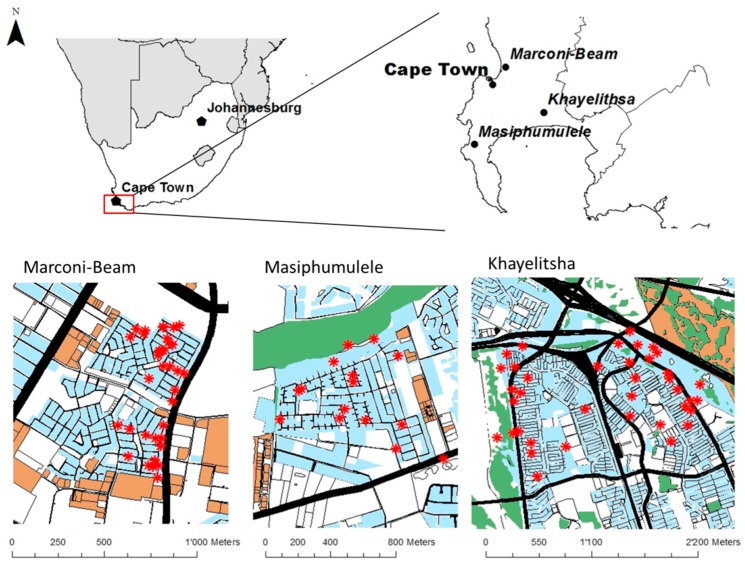
Overview of the three monitoring areas, Western Cape, South Africa. The measurement sites are represented with red stars, the roads as black lines, urban area in light blue, industrial area orange, and vegetation in green.

This study was performed in the Western Cape Province, located in the south-western part of South Africa. It covers about 130 km^2^ and contains 6 million inhabitants, of which about 2 million live in the Cape Town area [18]. The population demographics comprise a large proportion of young adults (20–30 years old), probably due to migration from other provinces. It is estimated that around 20% of the population in the province live in informal settlements or other forms of informal housing. The number of informal dwellings in Cape Town between 2001 and 2011 increased by over 300,000, reflecting the general population growth in this region [19]. Three informal settlements (Khayelitsha, Marconi-Beam near Milnerton, and Masiphumulele near Noordhoek) were selected (see Figure 1) in the epidemiological study to represent areas with relatively high pollutant levels (Khayelitsha and Marconi-Beam) and low air pollution levels (Masiphumulele) as inferred from annual government reports [6]. All three informal settlements are comparable in terms of population demographic characteristics and socio-economic status.

### 2.2. Measurements

Locations for the NO_2_ and PM_2.5_ air pollution monitoring campaign were selected from the 600 home addresses of the participants in the health study, from which 43 were selected in Khayelitsha, 36 in Marconi-Beam, and 16 in Masiphumulele. The monitoring locations were identified so as to represent the full range of expected air pollution emissions based on three categories of proximity to streets. Sites were classified as proximity to roads (less than 50 m from a main road, 60% of sites), intermediate (50–100 m from a main road, 30% of sites) or urban background (more than 100 m from a main road, 10% of sites). Measurements were performed by trained fieldworkers in these locations as well as in one school in Marconi-Beam and Masiphumulele, two schools in Khayelitsha and at the official air pollution monitoring station in Khayelitsha. The selected sites were additionally monitored for noise, which led to predictive models of noise levels for the study participants, as described by Sieber et al., (2017) [20].

NO_2_ was measured using passive gas samplers (from Passam AG, Switzerland) [21], while PM_2.5_ was measured using “Integrated PM_2.5_ Mass Filters” composed of a Teflon filter connected to a vacuum pump by tubing and a size selective centrifugal cyclone. The pumps were programmed to run for 15 min per hour leading to a single PM_2.5_ weekly measurement per site. For both pollutants, quality was controlled by deploying blank and duplicate samplers in each season and study area. The measurement campaign lasted from November 2015 to March 2016 (warm season) and from June to September 2016 (cold season). The transition from warm to cold season was defined based on the sudden change in weather and wind direction at the end of March 2016 (predominantly oriented to the south in the warm season and to the north-west in the cold season). NO_2_ and PM_2.5_ were measured twice (once in each season) for a one-week period at each home or for a maximum of four consecutive weeks at the schools in Khayelitsha and Marconi-Beam, as well as at the Khayelitsha monitoring station. Thereafter, the samples were collected, stored in a refrigerator, and sent to the manufacturer in cooling boxes for analysis. During the site visit, the geographical coordinates of the sites were recorded using a GPS device.

### 2.3. Geographical Predictor Data and Local Sources

Previous studies have shown that the most important predictors of NO_2_ and PM_2.5_ LUR models are traffic-related variables, including distance to roads and traffic counts as well as land use data, population, and topographical information [12].

Geographical information was provided by the City of Cape Town for the three study areas. Some incomplete features (households, road categorization) were manually added using “OpenStreetMaps” visualization [22]. The collected datasets were also re-categorized for harmonization between areas. Road networks were categorized into two groups: major roads and smaller roads, based on assumed magnitude of traffic density. Further predictors were collected, including airports, bus routes, bus stops, taxi routes, dwellings, distance to coast, and land use. The land use data were split into nine categories; residential area, commercial area, industries, parks and open spaces, vegetation, water bodies, public areas, and restaurants. The Normalized Difference Vegetation Index (NDVI) at a 30 by 30 m resolution from the “U.S. Geological Survey” was also collected [23]. NDVI is an index for vegetation density obtained by satellite remote sensing and based on light absorption on the surface of the earth, that ranges from −1 to +1 (low to high density).

A separate protocol was developed for collection of specific point sources of air pollution, which are generally informal and therefore not accounted for in the usual GIS datasets and which could explain part of the spatial variation of NO_2_ and PM_2.5_. These additional sources were collected by visiting the three areas of interest, following a predefined itinerary. Information was collected on specific air pollution sources, together with their respective geographical coordinates, such as informal grills, waste collection or burning sites, gas stations, and construction sites. The main GIS predictors collected are summarized in Table 1.

In the Geographical Information System (GIS), buffer zones of 25, 50, 100, 300, 500, and 1000 m radii were drawn around each measurement site. Point, line, and area predictor data, such as population, roads, and land use, were intersected with the different buffers and respectively the sum of the number of points, length, and area were calculated within each buffer for each site. In addition, the distance to the nearest line feature was calculated. Buffered averages of NDVI at the individual measurement locations at 30, 100, 150, 200, 500, and 750 m were also calculated. The predictor variables were then exported and integrated to the final database. Inverse distance and inverse squared distance were calculated for all distance variables.

### 2.4. Temporal Adjustment

Due to a limited amount of monitoring equipment, NO_2_ and PM_2.5_ measurements took place at a maximum of 10 sites simultaneously. To calculate warm season, cold season, and annual (both warm and cold seasons) means of NO_2_ and PM_2.5_ at each site, the temporal variability in air pollution was accounted for using a method described in the exposure assessment manual from the ESCAPE study [24]. The air pollution monitoring station from the Cape Town international airport (Airport Company South Africa-ACSA monitoring station) was selected as the reference site for temporal adjustment of the measurements. The ACSA site was located between the three study areas (within 10 to 30 km) and had a near complete record of pollution and meteorological measurements during our study period, measuring PM_10_ hourly averages, solar radiation, and temperature for 2015 and 2016. The PM_10_ daily average was calculated if more than 25% of the hourly means were available for a day (for 95% of the days, more than 75% (18 h) of measurements were available). For days with less than 25%, the daily PM_10_ value was estimated as the mean between the previous and next available PM_10_ daily concentrations. Daily PM_2.5_ means were estimated as 50% of the PM_10_ daily concentration, as suggested from the literature [25,26]. NO_2_ hourly averages were only available from 2015 to mid-January 2016. For the remaining time period in 2016, NO_2_ hourly data was estimated using the association between NO_2_ and PM_10_ and solar radiation levels [27]. The correlation between NO_2_ levels measured and estimated using PM_10_ and solar radiation was 0.82 over the 2015 available data (daily NO_2_ = 17.35 + daily PM_10_ − 0.07 daily solar radiation).

From the measured and calculated NO_2_ and PM_2.5_ daily means at the reference station, weekly averages were calculated, corresponding to the individual measurement periods at each site. For each weekly measurement period a correction factor was calculated as the difference between the measurement and the seasonal mean (annual, warm season, cold season) at the reference site. This correction factor was then subtracted from our measurements to get the final temporally adjusted seasonal mean for each measuring site. For the sites with repeated measurements, an average was calculated to obtain a single estimation of warm season pollution concentration per site.

### 2.5. LUR Modelling

The LUR method as used in the ESCAPE project was used for the predictor selection. In summary, a supervised forward linear regression procedure was performed testing all predictors with non-null values for more than 10% of the dataset and with a cut-off criterion of at least 1% increase in R^2^. Between each step, the chosen predictors were verified, allowing only predictors with a coefficient having the sign in the expected direction of effect. The final models were also tested for correlation between the predictor variables (Variable Inflation Factor (VIF) <3), for significance (coefficients’ *p*-value less than 0.1) and for potential highly influential sites (Cook’s D <1).

All modelling was performed using the statistical software RStudio 3.2.2. In total six LUR models were developed for each pollutant (NO_2_ and PM_2.5_) and each season (warm, cold, and annual), pooling the measurement data from all three areas (Khayelitsha, Marconi-Beam, and Masiphumulele).

### 2.6. Validation

The internal validity of the six models was tested using a leave-one-out-cross-validation (LOOCV) method. Each monitoring site was removed and the model’s parameters were estimated using the n-1 remaining sites. The process was repeated for each site and the final validation R^2^ was calculated from the observed (seasonal means) and predicted values [28,29]. Additionally, the root mean square error (RMSE) and normalized mean bias (NMB) were computed for each model to get an indication of the prediction error. The models were also tested for spatial autocorrelation using the Moran’s I statistic (*p*-value greater than 5%).

## 3. Results

### 3.1. Measurements

NO_2_ and PM_2.5_ were measured at 95 locations (43 in Khayelitsha, 36 in Marconi-Beam, and 16 in Masiphumulele). Overall, 106 NO_2_ measurements (including repeated measurements at selected locations) were available for the warm season and 100 for the cold season. Eight measurements were missing due to lost samples or samples that could not be attributed to a specific location. One outlier measurement was excluded from the warm season. Eventually, NO_2_ data was available for 94 and 86 sites for warm and cold seasons respectively.

There were 102 PM_2.5_ measurements that were available for the warm season and 95 for the cold season. The reasons for the loss of some measurements availability are similar to that for NO_2_. For the warm season, seven measurements were excluded for technical reasons (pump dysfunction, insufficient, flooding, running time, missing sampler) and there were two outliers. For the cold season, 11 measurements were excluded for technical reasons and two outliers were excluded. Eventually, PM_2.5_ data was available for 84 and 75 locations for warm and cold seasons respectively.

### 3.2. Temporal Adjusted NO_2_ and PM_2.5_ Values

**Figure 2:**
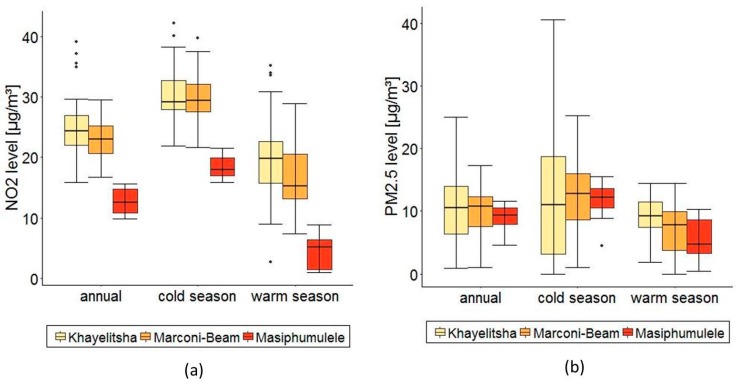
(**a**) Distribution of NO_2_ seasonal means in the three study areas, including median distribution, interquartile range (IQR) and whiskers (1.5 IQR); (**b**) Distribution of PM_2.5_ seasonal means in the three study areas, including median distribution, interquartile range (IQR) and whiskers (1.5 IQR).

After temporal adjustment, NO_2_ annual averages ranged between 9.9 μg/m^3^ and 39.1 µg/m^3^ with a mean of 22.1 μg/m^3^. NO_2_ levels were lower during the warm season (16.0 (9.6–20.9) μg/m^3^) compared to the cold season (27.9 (23.4–32.1) μg/m^3^), (see Supplementary Table S1). NO_2_ levels were highest in Khayelitsha and Marconi-Beam and lowest in Masiphumulele (see also Figure 2a).

After temporal adjustment, PM_2.5_ annual averages ranged between 0.9 μg/m^3^ and 25 μg/m^3^ with a mean of 10.2 μg/m^3^. PM_2.5_ levels were slightly lower in the cold than the warm season. The cold season demonstrated the widest range of PM_2.5_ levels, especially in Khayelitsha, between 0 and 40.7 μg/m^3^. Four negative values were set to zero (one in the warm season, three in the cold season). The highest values were observed in Khayelitsha for the warm season and in Marconi-Beam for the cold season. Annual PM_2.5_ values were similar for all three areas around 10 μg/m^3^ (also see Figure 2b).

The mean NO_2_ concentration at the reference station was 12.6 μg/m^3^ during the warm season, 24.2 μg/m^3^ during the cold season, and 18.4 μg/m^3^ over the entire year. PM_10_ mean concentrations were 24.9 μg/m^3^ for the warm season, 28.9 μg/m^3^ for the cold season, and 26.9 μg/m^3^ for the entire year. Correlations between adjusted and unadjusted warm season, cold season, and annual means were respectively 0.88, 0.86, and 0.93 for NO_2_ and 0.74, 0.94, and 0.91 for PM_2.5_. Compared to unadjusted measurements, adjusted warm season NO_2_ levels were somewhat higher in Khayelitsha (mean 19.8 vs. 16.0 μg/m^3^) and somewhat lower in Masipumulele (mean 4.5 vs. 6.5 μg/m^3^). The opposite was observed for the cold season. PM_2.5_ warm season mean adjusted levels increased in Khayelitsha and decreased in Marconi-Beam. For the cold season, the levels remained stable except in Masiphumulele where they increased after temporal adjustment (mean 11.6 μg/m^3^ vs. 7.2 μg/m^3^).

### 3.3. NO_2_ and PM_2.5_ LUR Models

Three LUR models were developed for each pollutant (see Table 2) for the combined three study areas (Khayelitsha, Marconi-Beam, and Masiphumulele). Supplementary Table S2 shows detailed information of the models including constant, coefficients, VIF, Cook’s D, and incremental R^2^. The annual NO_2_ LUR model explained 76% (CV; R^2^ = 0.72) of the spatial variability in the NO_2_ adjusted concentrations, 62% (CV; R^2^ = 0.57) for the warm season and 77% (CV; R^2^ = 0.72) for the cold season. The main predictors in the NO_2_ models included transportation variables (proximity to major roads for the warm season and annual models and proximity to bus stops or routes) for all three models. Additionally, the warm season model included the surface of transportation land use within 1000 m as a predictor. Proximity to refuse transfer stations was also an important NO_2_ predictor in all three models, as was proximity to grills for the cold season and annual models. Finally, the cold season model also included the proximity to the airport and number of dwellings within 1000 m.

The PM_2.5_ models were based on 91, 84, and 75 sites for annual, warm season, and cold season respectively, based on all three study areas. The PM_2.5_ LUR models explained 29%, 36%, and 29% of the spatial variability in the PM_2.5_ adjusted concentrations, for the annual, warm, and cold season respectively. The cross-validation for the annual, warm, and cold season yielded a R^2^ of 0.21, 0.26, and 0.19 respectively. The main predictors for PM_2.5_ included population density and distance to waste burning sites in all three models. Models for the cold season and annual PM_2.5_ levels also included proximity to construction sites, number of dwellings, and length of bus routes whereas the warm season model included the proximity to railways and grills.

RMSE and NMB values ranged between 2.9 and 4.8 (μg/m^3^) and between −3.1 × 10^−3^ and −3.9 × 10^−16^ respectively for the NO_2_ models and for the PM_2.5_ models between 3.1 and 7.1 (μg/m^3^) and between 6.4 × 10^−17^ and 3.8 × 10^−16^ respectively. Neither spatial auto-correlation nor influential sites were identified. For more information on the extent of the selected geographical predictors, please refer to Supplementary Table S3.

For both pollutants, the land use “water bodies” were excluded due to incomplete and suspected incorrect information.

### 3.4. Validation and Maps

**Figure 3:**
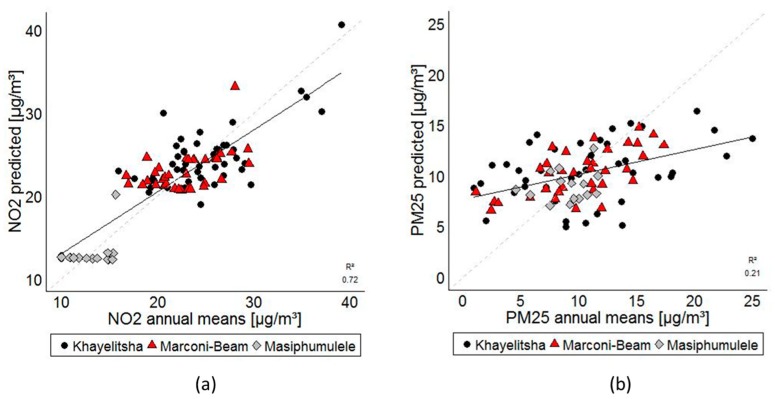
(**a**) Validation of NO_2_ predicted values against NO_2_ annual means. Scatter plot based on the results of leave-one-out-cross-validation (LOOCV), by study area; (**b**) validation of PM_2.5_ predicted values against PM_2.5_ annual means. Scatter plot based on the results of LOOCV, by study area. The 1:1 relationship between measured and predicted values is presented as a dotted line.

**Figure 4:**
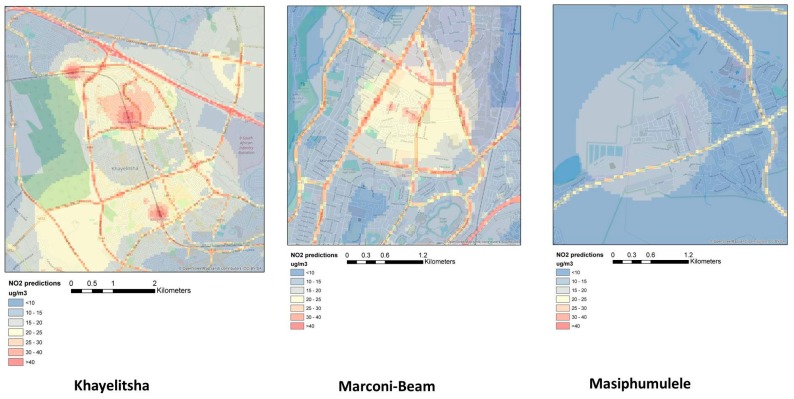
Predictive maps of annual NO_2_ levels in all three study areas based on the annual land use regression (LUR) model.

Figure 3a,b presents the scatter plots of the LOOCV between NO_2_ and PM_2.5_ predicted and adjusted annual mean values. Both models slightly overestimate the low pollution concentrations and underestimate the higher values. Figure 3a also shows that the model fit is driven by Khayelitsha and Marconi-Beam, and that the LUR model is unable to predict the variation in Masipumulele. Figure 4 presents the predicted levels of NO_2_ in the Khayelitsha region using the annual LUR model.

## 4. Discussion

Few studies in Africa have attempted to model air pollution exposures at a small spatial scale and to our knowledge, this is the first one attempting to model outdoor air pollution levels in informal settlements [11]. Annual and seasonal land use regression models were developed for NO_2_ and PM_2.5_ for the three informal settlements (Khayelitsha, Marconi-Beam, and Masiphumulele) in the Western Cape province of South Africa. Strong LUR models were developed for NO_2_, explaining between 62% and 77% of the variance. PM_2.5_ LUR models performed less well, explaining only between 29% and 36% of the overall variance. All models developed were robust with LOOCV R^2^’s similar to the models R^2^’s.

The adjusted annual mean NO_2_ values were low in all three study areas compared to the WHO annual mean NO_2_ reference guideline of 40 μg/m^3^ [2]. NO_2_ levels were considerably lower in Masiphumulele as compared to Khayelitsha and Marconi-Beam, with an average adjusted NO_2_ annual mean of 12.7 μg/m^3^. Masiphumulele is located in the most western part of the Cape Peninsula close to the coast and some distance away from the busy traffic areas of Cape Town and naturally yields lower air pollution levels. Khayelitsha and Marconi-Beam, located within the higher urbanization zone, have almost twice the NO_2_ levels of Masiphumulele (average adjusted NO_2_ annual means of respectively 25 and 23 μg/m^3^).

During the cold season, measured NO_2_ levels were higher in all three areas compared to the warm season and higher in Marconi-Beam than in Khayelitsha. An oil refinery, one of the probable main sources of NO_2_ in Marconi-Beam [30] was not in function during the warm season measurements, which could explain part of the observed trend. In addition, average wind speed in Cape Town is higher during the warm season, dispersing air pollution and thus yielding lower levels. The opposite occurs during the cold season, when lower average wind speed results in air remaining stagnant causing higher pollution levels (monthly wind speed of 3 m/s in cold month and 6 m/s in warm month have been recorded at the airport reference station). Similar patterns observed in NO_2_ levels were found in the PM_2.5_ measurement data. Annual PM_2.5_ concentrations were low, although in all three areas some sites had PM_2.5_ levels above the WHO air quality guideline for PM_2.5_ annual mean of 10 μg/m^3^. Masiphumulele again had the lowest measured levels, although not as low compared to Khayelitsha and Marconi-Beam, as was observed in the NO_2_ data. Seasonal variability in the PM_2.5_ measurements demonstrated, as for NO_2_, higher levels during the cold season with Masiphumulele and Marconi-Beam yielding higher average cold season means (11.7 μg/m^3^) than Khayelitsha (12.5 μg/m^3^), although the range in the latter is much wider (0 to 41 μg/m^3^). This wider range can be explained by the higher extent of Khayelitsha area as compared to Marconi-Beam and Masiphumulele and higher heterogeneity of the fine particle predictors in this area.

The seasonal variations observed in our measurement data reflect similar results from previous studies conducted in Cape Town with generally higher pollutant levels during the cold months, especially for NO_2_ with mean values around 22 μg/m^3^ and 30 μg/m^3^ for the warm and cold season respectively [31].

The annual NO_2_ LUR model explained a large component of the spatial variability (76%), which is comparable to other studies of annual NO_2_ LUR models, such as for example in Europe (median R^2^ of 0.82 across 36 study areas) [28], in California, US (R^2^ 0.71), Toronto, Canada (R^2^ 0.69) [32], and Taiwan (R^2^ 0.74) [33]. A recent study in Durban in the KwaZulu-Natal province of South Africa also developed a NOx LUR model explaining 73% of variance [16]. However, very few studies have developed seasonal models. A study in Antwerp, Belgium also produced annual, cold, and warm season NO_2_ LUR models explaining respectively 87%, 86%, and 84% of the variance [34]. Traffic is one of the main sources of high NO_2_ [10] and this is reflected in the traffic related predictors present in all three models, including proximity to major roads, bus stops and routes, and area of transportation land use. Traffic related variables were also present in the NO_2_ models in the above mentioned studies. Other variables that remained in the models were distance to refuse transfer station and proximity to grills, the latter variable demonstrating the importance of including local cooking sources, not well captured by routine GIS data. More generally, the selected monitoring site locations appeared to present high diversity in terms of concentrations and predictors, ranging from a background area (Masiphumulele) to more traffic exposed sites (Khayelitsha). This variability was relatively well captured by the model of the current study, as shown by the adjusted R^2^ (62% to 77%). However, less variation of NO2 was observed within Masiphumulele compared to the other two areas and this variation was not well captured with the models’ selected predictors. As the measured NO_2_ values were generally lower in this area as compared to the two others, they served as background values to fit the model. The general robustness of the model is indicated by a minute drop in the marginally lower LOOCV R^2^ and by stable predictor variables (low VIF and Cook’s D).

In contrast to the annual NO_2_ LUR model, the annual PM_2.5_ LUR model could only explain 29% of the variance. Though other studies have found mixed results in explaining the spatial variability of PM_2.5_, such as Pearl River Delta, China (R^2^ = 0.88) [35], Europe (median R^2^ = 0.71 across 20 study areas), Los Angeles, USA (R^2^ = 0.69) [36], and the Netherlands (R^2^ = 0.57), the validity of our model was substantially lower [37]. As with other studies, population or housing density appeared to be a good predictor for fine particulates [12]. Small, local waste burning sites, many of them of an informal nature, explained a fraction of the variability in PM_2.5_ in all three models. The number of grills within a 1000 m radius impacted on PM_2.5_ levels in the warm season only, which could be explained by the seasonality of outdoor grilling. Bus routes were also good predictors of PM_2.5_ concentrations, possibly due to the fact that buses in Cape Town run predominantly on diesel, which is a well-known source of fine particulates. Finally, construction sites within a 100 m radius remained in the annual and cold season models, possibly due to dust from construction sites being blown by the wind. The collected local sources seemed to account for an important part of the PM_2.5_ observed variability. The partial lack of such sources and their potentially transient nature could explain the lower performance of the PM_2.5_ models. Since these sources were only identified at one point in time, they do not take into account temporal variability and are generally difficult to capture. In particular, seasonal practices such as sitting around open fires during cold months, often burning plastic fuels, were not taken into account in the present study and could explain some additional variability in the data, as well as the higher pollution levels during the cold season. Another reason for the poor PM_2.5_ models can be attributed to the lower overall variability in measured PM_2.5_ compared to NO_2_ (which was to be expected, as PM_2.5_ is a regional pollutant). The fine particulate levels may be influenced much more by meteorological factors, such as the wind, which is particularly strong in the Cape Town area. Finally, the study areas were rather small. Some additional variance could be specific to certain study areas and better captured with individual models if the study areas were big enough.

While other air pollution modelling methods are available to model spatial variation of NO_2_ and PM_2.5_, such as spatial interpolation or dispersion models, they either lack precision or demand large amounts of data, making them less attractive for exposure mapping of large populations. LUR models have gained in popularity since they offer high resolution and describe spatial variability with high precision, even though their application area is restricted locally to the surface area covered by the measurements [13]. The spatial variation captured by the LUR models also helps in reducing exposure misclassification often observed when exposure estimates in a population are directly derived from one neighboring monitoring station. This is particularly important in urban areas, where the spatial variability is especially high for NO_2_ levels typically decreasing two to three fold within 50–100 m from the road [1].

The choice of the reference monitoring station for temporal adjustment represented one of the big challenges of this study. Adjusted means are generally calculated for each area using continuous monitoring data from a monitoring station within the study area. Such reference sites were however not available for all the areas and when available, NO_2_ and PM_2.5_ data were not available for the specified time period. The airport monitoring station was selected as an acceptable alternative, having: (1) daily PM_10_ measurements available for the entire time period; (2) daily NO_2_ measurements available for part of the time period; and (3) its location in close proximity to the three informal settlements (10 to 30 km). However, the resulting imprecisions obtained in the calculation of adjusted means could have affected the power of the model. Furthermore, temporal adjustment with a differential correction factor (as opposed to a ratio) can always be subject to underestimation, especially when levels were low as it is the case in this study. Although not ideal in terms of data availability, this study presents an approach to perform temporal adjustment when monitoring data is partly missing, which is a reality in many situations.

## 5. Conclusions

LUR modelling has been developed and used mainly in European and North American countries to adequately describe the spatial distribution of air pollution in urban settings with high spatial resolution. It is typically used to predict industrial and traffic-related pollutants such as NO_2_, particulate matter and ultrafine particles. The sources and spatial distribution of these pollutants can be very different in African countries. Despite the challenges faced in terms of data availability and reference measurements, this study was able to develop NO_2_ LUR models, which will be used to study exposure response relationships for asthma among school children in these informal settlements. The rather poor model performance of PM_2.5_ underscores the notion of possibly fundamental differences in the spatial determinants of particles in this African context. Thus, applicability to health studies may be limited and further research is needed to better understand the spatial patterns and determinants of PM_2.5_ concentrations in these areas of South Africa.

## Figures and Tables

**Table 1:** List of the main GIS predictors collected and used for predictive land use regression (LUR) models of NO_2_ and PM_2.5_ concentrations, including buffer size, unit, transformations and expected direction of the effect.

Category	GIS Variable Name	Variable Description	Unit	Buffer Radius (m)	Expected Effect
Roads	MAJROAD	Length of major roads	m	25/50/100/300/500/1000	+
	MAJROAD_d	Distance to nearest major road	m^−1^; m^−2^		+
	ROAD	length of roads (all)	m	25/50/100/300/500/1000	+
	ROAD_d	Distance to nearest road (all)	m^−1^; m^−2^		+
Taxi	TAXI	Length of taxi routes	m	25/50/100/300/500/1000	+
	TAXI_d	Distance to nearest taxi route	m^−1^; m^−2^		+
Bus	BUS_RTE	Length of bus routes	m	25/50/100/300/500/1000	+
	BUS_ST_c	Bus stops	#	25/50/100/300/500/1000	+
	BUS_ST_d	Distance to nearest bus stop	m^−1^; m^−2^		+
Rail	RAIL	Length of railways	m	25/50/100/300/500/1000	+
	TRAINSTAT	Distance to nearest train station	m^−1^; m^−2^		+
Airport	AIR	Distance to nearest airport	m^−1^; m^−2^		+
Point sources	BURN_c	Waste burning sites	#	25/50/100/300/500/1000	+
	BURN_d	Distance to nearest waste burning sites	m^−1^; m^−2^		+
	GRILL_c	Open grills	#	25/50/100/300/500/1000	+
	GRILL_d	Distance to nearest open grill	m^−1^; m^−2^		+
	CONSTRUCTION	Construction sites	#	25/50/100/300/500/1000	+
	REFTSTAT_d	Distance to nearest refuse transfer station	m^−1^; m^−2^		+
Population	INFORMAL	Area of informal settlements	m^2^	25/50/100/300/500/1000	+
	ORIGDWELL	Population/building density	#	25/50/100/300/500/1000	+
	ALLDWELL	Population/building density (from a different source, including informal housings)	#	25/50/100/300/500/1000	+
Land use	LU1	Residential	m^2^	25/50/100/300/500/1000	+
	LU2	Commercial	m^2^	25/50/100/300/500/1000	+
	LU3	Industrial	m^2^	25/50/100/300/500/1000	+
	LU4	Open space	m^2^	25/50/100/300/500/1000	−
	LU5	Vegetation	m^2^	25/50/100/300/500/1000	−
	LU6	Water bodies	m^2^	25/50/100/300/500/1000	−
	LU7	Public places	m^2^	25/50/100/300/500/1000	+
	LU8	Transportation	m^2^	25/50/100/300/500/1000	+
	LU9	Restauration	m^2^	25/50/100/300/500/1000	+
Vegetation	NDVI	Normalized Difference Vegetation Index	−1 to +1	30/100/150/200/500/750	−
Coast	COAST	Distance to coast	m^−1^; m^−2^		−

**Table 2:** Description of the NO_2_ and PM_2.5_ final models for each season based on the three study areas. Includes the list of best predictors, models’ summary statistics, and validation’s statistics.

Poll.	Season	Predictors	Model			LOOCV*			
			R^2^*	RMSE*	NMB*	R^2^*	RMSE*	NMB*	N*
**NO_2_**	**Warm**	LU8_1000 + MAJROAD_d + BUS_ST_d + REFSTAT_d + BUS_STOP_500	**0.62**	4.8	−9.9 × 10^−16^	**0.57**	5.1	−2.5 × 10^−3^	94
	**Cold**	GRILL_d + AIR_d + ALLDWELL_1000 +BUS_RTE_300 + REFSTAT_d + BUS_RTE_d	**0.77**	2.9	−3.1 × 10^−3^	**0.72**	3.2	−2.1 × 10^−4^	85
	**Annual**	MAJROAD_d + BUS_ST_d + GRILL_100 + REFSTAT_d + GRILL_1000 + TRAINSTAT	**0.76**	2.9	−3.9 × 10^−16^	**0.72**	3.1	2.5 × 10^−4^	97
**PM_2.5_**	**Warm**	RAIL_1000 + GRILL_d + ORIGDWELL_50 + BURN_d + GRILL_500 + REFSTAT_d	**0.36**	3.1	6.4 × 10^−17^	**0.26**	3.3	−2.1 × 10^−4^	84
	**Cold**	ALLDWELL_300 + CONSTRUCTION_100 + ORIGDWELL_25 + BUS_RTE_300 + BURN_d	**0.29**	7.1	1.5 × 10^−16^	**0.19**	7.6	−5.4 × 10^−3^	75
	**Annual**	ALLDWELL_300 + CONSTRUCTION_100 + ORIGDWELL_25 + BURN_d + BUS_RTE_300	**0.29**	4.0	3.8 × 10^−16^	**0.21**	4.3	−1.8 × 10^−3^	91

*LOOCV: Leave-One-Out-Cross-Validation: the robustness of the model is tested by successively taking one observation out of the sample, fitting the model on the remaining observations and testing its predictive performance (R^2^) on the observation left aside and repeating the process for each observation; *N: Number of sites; *R^2^: Coefficient of determination (R squared); *RMSE: Root-mean-square-deviation; *NMB: Normalized mean bias.

## Supplementary Materials

The following are available online at: http://www.mdpi.com/1660-4601/15/7/1452/s1. Table S1: Distribution of NO_2_ and PM_2.5_ seasonal means over the three study areas. Table S2: List of the 6 LUR models for each season (warm season, cold season, overall year) and each pollutant (NO_2_ and PM_2.5_). The best predictors for each model are listed, together with their respective coefficients, standard error (SE), and incremented R². Details of the models statistics are listed as well. Table S3: Summary statistics of the GIS predictors selected for the six LUR models, including minimum and maximum values, mean values, and percentiles distributions).
