# Supplementary material for: Land Use Regression Modelling of Outdoor NO2 and PM2.5 Concentrations in Three Low Income Areas in the Western Cape Province, South Africa
Source: Int J Environ Res Public Health. 2018 Jul 10;15(7):1452. doi: 10.3390/ijerph15071452 (PMC6069062; doi:10.3390/ijerph15071452)
Supplement: Supplementary file 1 [file ijerph-15-01452-s001.pdf]

Supplementary Materials

Table S1: Distribution of NO_2 and $PM_{2.5}$ seasonal means over the three study areas

	NO_2			$PM_{2.5}$		
	Warm season[1]	Cold season[2]	Annual	Warm season[1]	Cold season[2]	Annual
**N. obs**	94	86	97	84	75	91
**Mean**	**16.0**	**27.9**	**22.1**	**7.7**	**12.2**	**10.2**
**Median**	16.4	28.6	22.7	8.1	12.5	10.0
**Min.**	1.0	15.9	9.9	0.0	0.0	0.9
**25th perc.**	9.6	23.1	19.1	4.6	5.0	7.3
**75th perc.**	20.9	32.1	26.0	10.2	16.1	12.7
**Max.**	35.2	42.1	39.1	14.5	40.7	25.0

[1] Warm season : October to March / [2] Cold season: April to September

Table S2: List of the 6 LUR models for each season (warm season, cold season, overall year) and each pollutant (NO_2 and $PM_{2.5}$). The best predictors for each model are listed, together with their respective coefficients, standard error (SE) and incremented R^2. Details of the models statistics are listed as well.

Pollutant	Season	Predictors	Coefficients (SE)	SE	sd	Incremented R^2	Model			LOOCV			N
							RMSE	R^2	Adj. R^2	RMSE	R^2	Adj. R^2	
NO_2	Warm season	(Intercept)	-0.52	0.17	1.66		4.8	0.62	0.59	5.1	0.57	0.57	94
		area_8_1000m	0.00	0.00	0.00	0.40							
		invsqu_majroads_dist	1647.04	36.15	350.44	0.49							
		inv_IRTstops_dist	339.02	15.02	145.65	0.54							
		inv_refstat_dist	11729.10	316.81	3071.59	0.58							
		cnt_500_IRTstops	0.32	0.01	0.11	0.62							
	Cold season	(Intercept)	17.44	0.08	0.75		2.9	0.77	0.75	3.2	0.72	0.71	85
		inv_f3_dist	82.05	2.42	22.27	0.08							
		invsqu_airport_dist	54236664.33	688774.91	6350190.92	0.10							
		cnt_1000_alldwell	0.00	0.00	0.00	0.58							
		irtroutes_chloemaisons300m	0.00	0.00	0.00	0.62							
		invsqu_refstat_dist	7183809.45	326432.33	3009557.39	0.63							
		invsqu_allbusroutes_dist	1387.17	21.84	201.33	0.77							
	Annual	(Intercept)	9.30	0.10	0.98		2.9	0.76	0.75	3.1	0.72	0.72	97
		invsqu_majroads_dist	1559.67	21.40	210.80	0.22							
		inv_IRTstops_dist	317.74	7.87	77.47	0.34							
		cnt_100_f3	0.75	0.03	0.28	0.37							
		invsqu_refstat_dist	10640943.97	294799.35	2903436.92	0.42							
		cnt_1000_f3	0.80	0.01	0.09	0.64							
		inv_trainstat_dist	2461.33	37.47	369.05	0.76							
$PM_{2.5}$	Warm season	(Intercept)	0.02	0.17	1.53		3.1	0.36	0.31	3.3	0.26	0.25	84
		railways_chloemaisons1000m	0.00	0.00	0.00	0.17							
		inv_f3_dist	58.12	3.37	30.92	0.20							
		cnt_50_alldwell	0.03	0.00	0.01	0.24							
		inv_w3_dist	1764.49	61.21	561.02	0.26							

		cnt_500_f3	0.55	0.02	0.17	0.32							
		inv_refstat_dist	4749.29	238.49	2185.80	0.36							
	**Cold season**	(Intercept)	-1.24	0.35	3.03		7.1	0.29	0.24	7.6	0.19	0.17	75
		cnt_300_adrjoin	0.01	0.00	0.00	0.15							
		cnt_100_c5	6.14	0.29	2.50	0.19							
		cnt_25_alldwell	0.21	0.01	0.09	0.23							
		irtroutes_chloemaisons300m	0.00	0.00	0.00	0.24							
		inv_w3_dist	2218.83	125.92	1090.48	0.29							
	**Annual**	(Intercept)	3.21	0.15	1.42		4.0	0.29	0.25	4.3	0.21	0.20	91
		cnt_300_adrjoin	0.01	0.00	0.00	0.12							
		cnt_100_c5	3.30	0.14	1.30	0.17							
		cnt_25_alldwell	0.16	0.01	0.05	0.23							
		invsqu_w3_dist	262695.48	13195.79	125879.80	0.24							
		irtroutes_chloemaisons300m	0.00	0.00	0.00	0.29							

Table S3: Summary statistics of the GIS predictors selected for the six LUR models, including minimum and maximum values, mean values and percentiles distributions).

predictor	Min.	1st Qu.	Median	Mean	3rd Qu.	Max.
Landuse 8/Transport (surface within 1000m)	172388.3	560223.2	586307.3	587833.5	706931.5	1010012
major roads (inverse squared distance)	1.40E-06	1.29E-05	4.59E-05	0.000566	0.000359	0.01
Bus stops (inverse distance)	0	0.000691	0.002894	0.003503	0.004943	0.030276
Bus stops (count within 500m)	0	0	2	4.896907	8	18
Grills (inverse distance)	0.001199	0.003999	0.006376	0.01105	0.011249	0.099081
Airport (inverse squared distance)	0	0	0	4.75E-08	1.19E-07	1.92E-07
All dwellings (count within 1000m)	3	8	54	6153.278	12237	20800
Bus routes (length within 300m)	0	0	995.3878	1099.995	1400.427	4573.212
Refuse stations (inverse squared distance)	0	0	0	5.21E-08	1.24E-07	8.99E-07
All bus routes (inverse squared distance)	1.10E-05	2.74E-05	9.25E-05	0.000621	0.000379	0.01
Grills (count within 100m)	0	0	0	0.597938	1	4
Grills (count within 1000m)	2	7	12	10.13402	13	14
Train station (inverse distance)	0	0	0	0.00067	0.001122	0.004237
railways (length within 1000m)	0	23.53953	1104.969	984.9359	1589.676	2341.727
All dwellings (count within 50m)	0	0	0	27.35052	58	133
Waste burning site (inverse distance)	0	0	0.000658	0.000744	0.001039	0.005375
Grills (count within 500m)	0	3	4	5.731959	10	13
Addresses (count within 300m)	65	558	719	742.1959	912	1738
Construction sites (count within 100m)	0	0	0	0.216495	0	3
All dwellings (count within 25m)	0	0	0	6.896907	14	48
